# Enhanced Absorption and Growth Inhibition with Amino Acid Monoester Prodrugs of Floxuridine by Targeting hPEPT1 Transporters

**DOI:** 10.3390/molecules13071441

**Published:** 2008-06-28

**Authors:** Yasuhiro Tsume, Balvinder S. Vig, Jing Sun, Christopher P. Landowski, John M. Hilfinger, Chandrasekharan Ramachandran, Gordon L Amidon

**Affiliations:** 1 Department of Pharmaceutical Science, College of Pharmacy, University of Michigan, 428 Church Street, Ann Arbor, MI 48109-1065, USA; E-mails: ytsume@umich.edu; rcmad@umich.edu; 2 Pharmaceutical Research Institute, Bristol-Myers Squibb Company, New Brunswick, NJ 08502; E-mail: balvinder.vig@bms.com; 3 Department of Medicinal Chemistry, University of Michigan, Ann Arbor, Michigan 48109, USA; Email: sunjing@umich.edu; 4 Institute of Biochemistry and Molecular Medicine, University of Bern, CH-3012 Bern, Switzerland; Email: christopher.landowski@mci.unibe.ch; 5 TSRL, Inc. Ann Arbor, Michigan 48108, USA; Email: jhilfinger@tsrlinc.com

**Keywords:** 5-FU, floxuridine, prodrugs, Caco-2 permeability, cell proliferation assays, oligopeptide transporter 1 (PEPT1)

## Abstract

A series of amino acid monoester prodrugs of floxuridine was synthesized and evaluated for the improvement of oral bioavailability and the feasibility of target drug delivery via oligopeptide transporters. All floxuridine 5′-amino acid monoester prodrugs exhibited PEPT1 affinity, with inhibition coefficients of Gly-Sar uptake (IC_50_) ranging from 0.7 – 2.3 mM in Caco-2 and 2.0 – 4.8 mM in AsPC-1 cells, while that of floxuridine was 7.3 mM and 6.3 mM, respectively. Caco-2 membrane permeabilities of floxuridine prodrugs (1.01 – 5.31 x 10^-6^ cm/sec) and floxuridine (0.48 x 10^-6^ cm/sec) were much higher than that of 5-FU (0.038 x 10^-6^ cm/sec). MDCK cells stably transfected with the human oligopeptide transporter PEPT1 (MDCK/hPEPT1) exhibited enhanced cell growth inhibition in the presence of the prodrugs. This prodrug strategy offers great potential, not only for increased drug absorption but also for improved tumor selectivity and drug efficacy.

## Introduction

The anti-metabolites 5-fluorouracil (5-FU) and floxuridine (5-fluoro-2'-deoxyuridine) continue to be mainstay drugs for colorectal cancer treatment after 50 years [1]. However, the improvement of 5-FU and floxuridine therapeutic efficacy is critical because of the poor response rate of 5-FU, erratic oral absorption of floxuridine, and the adverse effects associated with those chemotherapeutics [2]. The anabolic mechanism of 5-FU and floxuridine is well studied [3]. Floxuridine activity is quite specific for DNA related pathways and results in DNA-directed cytotoxicity with little or no RNA directed cytotoxicity, unlike 5-FU [4,5,6]. The utilization of the more potent floxuridine is appealing because it inhibits *in vitro* cell proliferation at 10- to 100-fold lower concentrations compared to 5-FU [7,8,9]. However, the abundant presence of thymidine phosphorylase (TP) in many tissues, including the liver and intestine, rapidly converts floxuridine to 5-FU [10]. Thus, improving the resistance of floxuridine to enzymatic degradation may also increase its therapeutic efficacy. With the consideration of improved chemical stability, the prodrugs must be converted to active compounds for the desired therapeutic effect. In prodrug development, the activation of the prodrug is an essential step. It has been suggested that the biphenyl hydrolase-like protein recently identified as being responsible for hydrolysis of the prodrug valacyclovir (VACVase), might be involved in the activation of other amino acid prodrugs [11]. Kim *et al*. suggest that the substrate specificity of this enzyme is largely determined by the amino acid acyl promoiety of prodrug [12]. Another enzyme, carboxylesterase I, has been shown to preferentially hydrolyze phenylalanine containing nucleoside ester prodrugs, while also displaying 100-fold less activity toward aliphatic esters [13].

Amino acid ester prodrugs of floxuridine and the antiviral agent acyclovir have been shown to be substrates of the PEPT1 transporter [14, 15]. PEPT1 has broad substrate specificity for dipeptides, tripeptides, and β-lactam antibiotics [16,17,18,19,20,21,22,23]. Improved oral bioavailability of the valyl ester prodrug of acyclovir has been attributed to the presence of oligopeptide transporters [24, 25]. Two pancreatic cancer cell lines, AsPC-1 and Capan-2, have been reported to have significant expression of oligopeptide transporters, where they might represent possible targets for cancer therapy [26]. Selective growth inhibition studies in *in vitro* cell systems exogenously expressing PEPT1 have demonstrated more accumulation of cancer drug in tumor cells for an enhanced therapeutic effect [15,27]. Those results support the notion that the promoieties that incorporate amino acids, dipeptides, and tripeptides are well recognized by PEPT1, PEPT2, and ATB^0+^ transporters [17, 28,29,30,31,32]. Thus, amino acid modification of cancer drugs represents a potential drug delivery strategy to target cells via transporters.

In this report, we briefly describe the synthesis and characterization of mono amino acid ester prodrugs of floxuridine. We evaluate the prodrug stability, Caco-2 membrane permeability and the feasibility of selective tumor growth inhibitory effect in MDCK and MDCK/hPEPT1 cells by cell proliferation assays.

## Results and Discussion

**Scheme 1:**
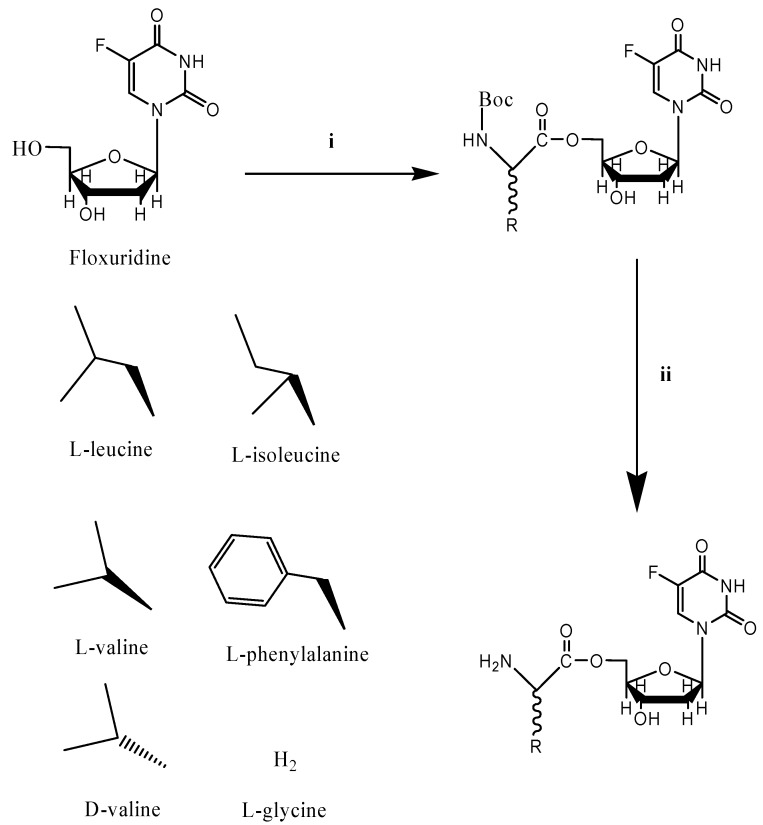
Synthesis of amino acid ester prodrugs of floxuridine.

Prodrug approaches with amino acid modification have been widely employed to improve intestinal absorption of poorly permeant drugs [33]. The antiviral drug valacyclovir is an example of a successful amino acid ester prodrug strategy [34]. The improved oral bioavailabiliy of valacyclovir has been attributed to the enhanced transport by intestinal oligopeptide transporters [14,24,35]. Dipeptide and tripeptide compounds, along with mono amino acid derivatives, have been investigated for their suitability as substrates for the oligopeptide transporter [16,17,18,21,28,36,37,38,39,18,21,28,36,37,38,39]. Mono amino acid ester prodrugs of antiviral and anticancer drugs such as gemcitabine, acyclovir, and 2-bromo-5,6-dichloro-1-(*β*-D-ribofuranosyl)benzimidazole (BDCRB) have been synthesized and evaluated for their suitability as transporter substrates in our previous reports [15,24,40,41,42,43]. Mono amino acid floxuridine prodrugs reported herein were synthesized as shown in Scheme 1. 

*Reagents:* (i) *N-t*-BOC-protected amino acids, DCC, DMAP, DMF; (ii) TFA, CH_2_Cl_2_

The total prodrug yields for each amino acid ranged between 2 and 18 % and the purity for all prodrugs was > 95 %, as determined by HPLC. The impurities were easily separated from their parent compounds by reverse-phase HPLC. All prodrug structures and identities were confirmed by ESI-MS and NMR. The prodrug purity and mass spectral data are shown in Table 1. 

**Table 1:** Analytical data for amino acid ester prodrugs of floxuridine.

	% Purity (HPLC)	ESI-MS (M + H)^+^		Molecular Weight (TFA salt)	log P^*^
		Required	Observed		
5'-O-L-leucyl-floxuridine	96.77	360.2	360.4	473.4	-0.95
5'-O-L-phenylalanyl-floxuridine	96.10	394.2	394.0	507.4	-0.51
5'-O-L-valyl-floxuridine	98.51	346.2	346.0	459.4	-1.30
5'-O-D-valyl-floxuridine	99.52	346.2	346.0	459.4	-1.30
5'-O-L-isoleucyl-floxuridine	95.96	360.2	360.4	473.4	-0.78
5'-O-L-glycyl-floxuridine	95.23	304.2	303.9	417.4	-2.68

* Calculated using ChemDraw 7.0.

The experiments concerning prodrug stability were performed at 37°C in pH 7.4 phosphate buffers and Caco-2, AsPC-1, and MDCK cell homogenates. Table 2 displays the estimated half-lives (t_1/2_) obtained from linear regression of pseudo-first-order plots of prodrug concentration vs. time for the floxuridine prodrugs in pH 7.4 phosphate buffers alone and in Caco-2, AsPC-1, and MDCK cell homogenates.

**Table 2:** Half-lives of the hydrolytic degradation of floxuridine prodrugs in pH 7.4 buffer, and in homogenates from Caco-2 cells, AsPC-1 cells, and MDCK cells.

	Half Life (min)			
Prodrug	Buffer pH 7.4	Homogenates from Caco-2 cells	Homogenates from AsPC-1 cells	Homogenates from MDCK cells
Floxuridine	nd	5.7 ± 0.3	6.4 ± 3.2	68.9 ± 12.8
5'-O-l-valyl-floxuridine	303.9 ± 17.8	9.4 ± 0.6	18.7 ± 6.7	74.7 ± 5.3
5'-O-d-valyl-floxuridine	344.9 ± 10.2	342.6 ± 120.2	290.9 ± 48.9	311.6 ± 45.4
5'-O-l-phenylalanyl-floxuridine	221.7 ± 56.7	11.1 ± 9.9	11.8 ± 1.7	6.0 ± 0.6
5'-O-l-leucyl-floxuridine	77.3 ± 1.2	4.8 ± 0.2	2.0 ± 0.1	9.2 ± 1.1
5'-O-l-isoleucyl-floxuridine	323.5 ± 1.5^a^	192.3 ± 31.8	198.0 ± 34.1	244.9 ± 18.3
5'-O-l-glycyl-floxuridine	85.5 ± 3.2	24.1 ± 2.0	27.6 ± 5.8	11.2 ± 2.0

Values are presented as mean ± S.D.; nd = not determined; ^a^from Ref. [40].

The 5ʹ-O-d-valyl-floxuridine prodrug exhibited the highest stability in all conditions and thus confirmed previous reported results [44, 45]. These results indicate that there does not appear to be any significant enzymatic component in the breakdown of the 5ʹ-O-d-valyl ester prodrug in cell homogenates. All prodrugs except 5ʹ-O-l-glycyl-floxuridine and 5ʹ-O-l-leucyl-floxuridine exhibited the same degree of stability in phosphate buffers. The phenylalanine monoester prodrug exhibited 20-fold less stability in cell homogenates compared to pH 7.4 phosphate buffers. This result suggests that the phenylalanine promoiety was recognized by enzymes more readily than other amino acid residues and its ester prodrugs were enzymatically degraded. This observation agrees well with our previous enzymatic hydrolysis results [13]. The stability of the 5ʹ-O-d-valyl, 5ʹ-O-l-phenylanalyl, 5ʹ-O-l-isoleucyl, and 5ʹ-O-l-glycyl prodrugs were comparable in both Caco-2 and AsPC-1 cell homogenates. On the other hand, 5ʹ-O-l-valyl and 5ʹ-O-l-leucyl prodrugs exhibited significantly different half-lives in each cell homogenate and were stable in MDCK cell homogenates. In Caco-2 cell homogenates, the 5ʹ-O-l-valyl ester prodrug displayed a 2-fold shorter half-life than in AsPC-1 cell homogenates, while 5ʹ-O-l-leucyl ester prodrug was hydrolyzed 2.4- and 4.7-fold more quickly in AsPC-1 cells homogenates than in Caco-2 and MDCK cell homogenates, respectively. The IC_50_ values of amino acid monoester prodrugs of floxuridine for an oligopeptide transporter determined using inhibition of Gly-Sar uptake in Caco-2 and AsPC-1 cells are summarized in Table 3.

**Table 3:** Concentrations of floxuridine and floxuridine prodrugs required to inhibit Gly-Sar uptake by 50 % (IC_50_) in Caco-2 and AsPC-1 cells.

	IC_50_ Value (mM)	
Prodrug	Caco-2 cell	AsPC-1 cell
Floxuridine	7.3 ± 1.6	6.3 ± 2.3
5'-O-l-valyl-floxuridine	1.0 ± 0.1	2.9 ± 0.4
5'-O-d-valyl-floxuridine	2.6 ± 0.1	4.8 ± 1.3
5'-O-l-phenylalanyl-floxuridine	2.1 ± 0.1	2.0 ± 0.1
5'-O-l-leucyl-floxuridine	2.0 ± 0.3	2.6 ± 0.2
5'-O-l-isoleucyl-floxuridine	0.7 ± 0.0	4.1 ± 1.8
5'-O-l-glycyl-floxuridine	2.3 ± 0.6	2.7 ± 0.5

Values are presented as mean ± S.D.

All floxuridine ester prodrugs exhibited greater inhibition of Gly-Sar uptake by for hPEPT1 than did the parent drug, floxuridine. 5ʹ-O-l-isoleucyl-floxuridine displayed the highest inhibition of oligopeptide transporters in Caco-2 cells (IC_50_, 0.7 ± 0.0 mM), whereas 5ʹ-O-l-phenylalanyl-floxuridine had the highest affinity in AsPC-1 cells (IC_50_, 2.0 ± 0.1 mM). Not surprisingly, the D-stereoisomer ester prodrug 5ʹ-O-d-valyl-floxuridine showed the least inhibition of Gly-Sar uptake in both cells (IC_50_, 2.6 ± 0.1 mM in Caco-2 cells, IC_50_, 4.8 ± 1.3 mM in AsPC-1 cells). 5ʹ-O-d-valyl-floxuridine inhibited the transporters 1.6-fold to 2.5-fold less than did 5ʹ-O-l-valyl-floxuridine in AsPC-1 cells and Caco-2 cells, respectively, but the monoester prodrug with an unnatural form of amino acid still inhibited to an extent similar to that of the parent drug. With the exception of 5ʹ-O-l-phenylalanyl-floxuridine, all monoester prodrugs of floxuridine exhibited better IC_50_ values in Caco-2 cells than in AsPC-1 cells, likely due to over-expression of PEPT1 transporters in AsPC-1 cells [26].

**Figure 1:**
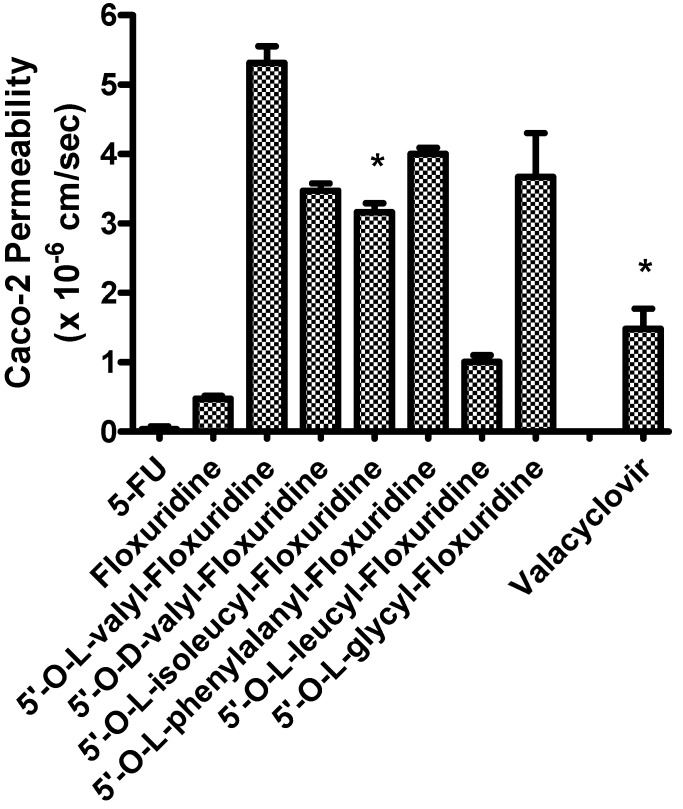
Caco-2 Permeability of 5-fluorouracil, floxuridine, and floxuridine prodrugs (Mean ± S.D.).

The apical-to-basolateral permeabilities of amino acid monoester prodrugs of floxuridine and their parent drugs, floxuridine and 5-FU, were determined at 37°C in Caco-2 cell monolayers. Figure 1 shows the membrane permeability values in Caco-2 cell system. Floxuridine, a prodrug form of 5-FU, showed a 12-fold higher Caco-2 membrane permeability than 5-FU. All floxuridine prodrugs exhibited 2- to 11-fold higher and 26- and 140-fold higher membrane permeability than floxuridine and 5-FU, respectively. Surprisingly, 5ʹ-O-d-valyl-floxuridine exhibited permeability that was as high as that of other amino acid ester prodrugs. No simple correlations between membrane permeability and transporter affinity in Caco-2 cells were evident in the prodrugs, even though our group previously reported excellent linear correlations between them in HeLa/PEPT1 cells [40]. The floxuridine amino acid monoester prodrugs, with the exception of 5ʹ-O-l-leucyl-floxuridine, demonstrated excellent membrane permeability in Caco-2 cells, better than that of a reference compound, valacyclovir (Figure 1). The detection of only 5-FU in the basolateral receiver compartment following transport of floxuridine across Caco-2 cell monolayers suggests the instability of glycosidic bond. Drug/prodrug stability in the mucosal cell would affect its drug membrane permeability and, therefore, the observation of low permeability of 5ʹ-O-l-leucyl-floxuridine among those prodrugs may be partly due to its rapid hydrolysis to floxuridine, 5-FU and beyond (Table 2).

The permeability values were calculated using the total amount of permeant drug (prodrug + floxuridine + 5-FU). * from Ref. [40].

The concentrations of floxuridine 5' amino acid monoester required for 50% growth inhibition (GI_50_) of MDCK cells with and without exogenous hPEPT1 (MDCK/hPEPT1) are shown in Table 4. The prodrug GI_50_ values are significantly lower in MDCK/hPEPT1 cells, compared to those in MDCK, indicating increased growth inhibition due to enhanced drug delivery by carrier-mediated transporters. The cell growth inhibition effect was improved 18-fold with the presence of hPEPT1 transporters when cells were treated with 5ʹ-O-L-isoleucyl-floxuridine. 5ʹ-O-L-leucyl-floxuridine exhibited the lowest GI_50_ values (2.3 µM in MDCK/hPEPT1 cells) and 10-fold greater growth inhibition with the expression of exogenous oligopeptide transporters. 5ʹ-O-L-valyl-floxuridine displayed about 6-fold enhancement in cell growth inhibition, while 5ʹ-O-d-valyl-floxuridine showed only 25% improvement of cell growth inhibition in MDCK/hPEPT1 cells. Floxuridine exhibited no enhancement (Enhancement Factor = 1.00) between MDCK and MDCK/hPEPT1 cells (data not shown). The lower observed effectiveness of 5ʹ-O-d-valyl-floxuridine in cell growth inhibition might be due to the prodrug stability in MDCK cells (Table 2). This observation indicates the necessity of prodrug activation for cancer cell growth inhibition and for the reduction in side-effects of the prodrug in non-tumor cells.

**Table 4:** Effect of hPEPT1 in MDCK Cells for Cell Proliferation Assay.

	GI_50_ (µM)		
Prodrug	MDCK	MDCK/hPEPT1	Enhancement Factor
5'-O- l-valyl-floxuridine	126.6 ± 7.7	21.1 ± 4.2	5.91
5'-O- d-valyl-floxuridine	88.5 ± 2.6	71.0 ± 2.7	1.25
5'-O- l-phenylalanyl-floxuridine	41.3 ± 3.9	4.7 ± 2.4	8.80
5'-O- l-leucyl-floxuridine	23.5 ± 3.0	2.3 ± 1.0	10.09
5'-O- l-isoleucyl-floxuridine	186.5 ± 4.2	10.3 ± 2.9	18.04
Values are presented as mean ± S.D.; The prodrug concentration required to inhibit growth by 50 % (GI_50_) was determined in MDCK cells and MDCK cells that overexpressed hPEPT1 (MDCK/hPEPT1). The ratios of GI_50_ in MDCK and MDCK/hPEPT1 cells are presented as Enhancement Factor.			

In all cases, the prodrug forms of floxuridine improved permeability in Caco-2 cells by as much as 11-fold over that of floxuridine and 140-fold over that of 5-FU. It was also demonstrated by MDCK cell growth inhibition assays that transporters could potentially serve as excellent delivery targets to improve chemotherapeutic efficacy.

## Conclusions

In summary, amino acid monoester prodrugs of floxuridine were synthesized and were demonstrated to be suitable substrates for a human PEPT1 transporter to improve oral absorption and the possibility of target drug delivery via transporters on target sites. All prodrugs exhibited better transport values than floxuridine and 5-FU. 5ʹ-O-d-valyl-floxuridine displayed good uptake inhibition of Gly-Sar and much less enzymatic breakdown in cell homogenates compared with 5ʹ-O-l-valyl-floxuridine. The prodrug stability in GI tract, blood, and organs such as the liver is a critical factor for successful oral drug delivery. From this point of view, 5ʹ-O-d-valyl-floxuridine exhibited 11 to 213-fold more enzymatic stability than the other prodrugs without compromising transporter affinity (IC_50_) and Caco-2 membrane permeability. Thus, 5ʹ-O-d-valyl-floxuridine may be the best candidate for *in vivo* oral bioavailability study.

## Experimental

### Materials

Floxuridine (Floxuridine) was obtained from Lancaster (Windham, NH, USA). The *tert*-butyloxycarbonyl (Boc) protected amino acids Boc-l-Leucine, Boc-l-Phenylalanine, Boc-l-Valine, Boc-d-Valine, Boc-l-Isoleucine and Boc-l-Glycine were obtained from Calbiochem-Novabiochem (San Diego, CA, USA). High-performance liquid chromatography (HPLC) grade acetonitrile was obtained from Fisher Scientific (St. Louis, MO, USA). 5-Fluorouracil (5-FU), *N,N*-dicyclohexyl-carbodiimide, *N,N*-dimethylaminopyridine, trifluoroacetic acid (TFA), and all other reagents and solvents were purchased form Sigma-Aldrich Chemical Co. (Milwaukee, WI, USA). Cell culture reagents were obtained from Invitrogen (Carlsbad, CA, USA) and cell culture supplies were obtained from Corning (Corning, NY, USA) and Falcon (Lincoln Park, NJ, USA). All chemicals were either analytical or HPLC grade.

### Floxuridine Prodrug Synthesis

The synthesis and characterization of 5ʹ-mono amino acid ester prodrugs of floxuridine have been reported previously [40, 43]. The l-glycyl prodrug of floxuridine was synthesized in a similar manner. Briefly, Boc-protected amino acids (Boc-l-glycine; 1 mmol), *N,N*-dicyclohexylcarbodiimide (1 mmol), and *N,N*-dimethylaminopyridine (0.1 mmol) were allowed to react with floxuridine (1 mmol) in dry DMF (7 mL) for 24 hours. The reaction progress was monitored by TLC (ethyl acetate). The reaction mixture was filtered and DMF was removed under vacuum at 40°C. The residue was extracted with ethyl acetate (30 mL) and washed with water (2 x 20 mL), and saturated NaCl (20 mL). The organic layer was dried over MgSO_4_ and concentrated under vacuum. The reaction yielded a mixture of 3'-monoester, 5'-monoester, and 3',5'-diester floxuridine prodrugs. The three spots observed on TLC were separated and purified using column chromatography (dichloromethane/methanol, 20:1). Fractions from each spot were concentrated under vacuum separately. The Boc group was cleaved by treating the residues with 1:1 TFA-dichloromethane (5 mL). After 4 hours, the solvent was removed and the residues were reconstituted with water and lyophilized. The TFA salts of amino acid prodrugs of Floxuridine were obtained as white fluffy solids. The combined yield, consisting of 3'-monoester, 5'-monoester, and 3',5'-diester floxuridine prodrugs of floxuridine, was 60%. Prodrugs were determined to be 95-99% pure by reverse-phase HPLC and were easily separated from their parent compounds by reverse-phase HPLC. The observed molecular weights of all prodrugs, determined by electrospray ionization mass spectra (ESI-MS) obtained on a Micromass LCT ESI-MS, were found to be consistent with those predicted by their structures. The structural identities of the prodrugs were confirmed using proton nuclear magnetic resonance spectra (^1^H-NMR) were obtained in DMSO-d_6_ on a 300 MHz Bruker DPX-300 NMR spectrometer.

*5'-L-glycyl-floxuridine*: yield, 8.5%; percent purity, 95%; ^1^H-NMR δ: 2.10-2.33 (2H, m, C2'), 3.80-3.97 (3H, m, α-CH_2_, C3'), 4.26 (1H, m, C4'), 4.38 (2H, d, *J* = 5.0 Hz, C5'), 6.17 (1H, t, *J* = 6.4 Hz, C1'), 7.95 (1H, d, *J* = 7.0 Hz, CHF); ESI-MS, 303.9 (M + H)^+^. 

### Cell Culture

AsPC-1 cells (passages 44-49, American Type Culture Collection, Rockville, MD, USA) were routinely maintained in RPMI-1640 containing 10% fetal bovine serum and Caco-2 cells (passages 30-55) and MDCK cells (Passages 35-40) (both also from the American Type Culture Collection) were routinely maintained in DMEM containing 10% fetal bovine serum, 1% nonessential amino acids, 1 mmol/L sodium pyruvate, and 1% L-glutamine. Cells were grown in an atmosphere of 5% CO_2_ and 90% relative humidity at 37°C and in antibiotic-free media to avoid the possible transport interference by antibiotics.

### Hydrolysis Studies

*Enzymatic Stability*. Confluent Caco-2 cells, AsPC-1 cells, and MDCK cells were rinsed twice with saline. The cells were washed with pH 7.4 phosphate buffer (5 mL, 10 mmol/L), lysed by ultrasonication (Micro ultrasonic cell disrupter Model KT40, Kontes, Vineland, NJ, USA), and pelleted by centrifugation for 5 minutes at 1,000 x g. Protein amount was quantified with the Bio-Rad (Hercules, CA, USA) DC Protein Assay using bovine serum albumin as a standard. The amount of protein was adjusted to 500 μg/mL and hydrolysis reactions were carried out in 96-well plates (Corning). Caco-2, AsPC-1, and MDCK cell suspensions (250 µL) were placed in triplicate wells, the reactions were started with the addition of substrate, and cells were incubated at 37°C for 120 minutes. At the desired time point, sample aliquots (35 µL) were removed and added to acetonitrile (ACN, 150 µL) with 0.1% TFA. The mixtures were filtered with a 0.45 µm filters at 1,000 x g for 10 minutes at 4°C. The filtrate was then analyzed via reverse-phase HPLC.

*Chemical Stability.* The nonenzymatic hydrolysis of the prodrugs was determined as described above, except that each well contained pH 7.4 phosphate buffers (10 mmol/L) instead of cell homogenate.

### Data Analysis

The initial rates of hydrolysis were used to obtain the apparent first-order rate constants and to calculate the half-lives. The apparent first-order degradation rate constants of various floxuridine prodrugs at 37°C were determined by plotting the logarithm of prodrug remaining as a function of time. The slopes of these plots are related to the rate constant, k, and given by


k = 2.303 × slope (log C vs. time)
(1)

The degradation half-lives were then calculated by the equation


t_1/2_ = 0.693/k (2)

Statistical significance was evaluated with GraphPad Prism v. 3.0 by performing one-way analysis of variance with post-hoc Tukey’s test to compare means.

The apparent permeability (*P_app_*) for the prodrugs was calculated using the following equation: 

*Flux* = *J _ss_* = *dM / dt* (3)

where J_ss_ is the steady state flux, M is the cumulative amount of prodrug, and regenerated mono amino acid prodrug, drug and 5-FU in the receiver compartment. The apparent permeability was calculated from steady state flux as follows:


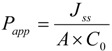
(4)

where A is the surface area of monolayer exposed to the permeant, C_0_ is the concentration of the prodrug in the donor solution. The concentrations of floxuridine and its prodrugs in the receiver and donor compartments were analyzed using HPLC.

### HPLC Analysis

The concentrations of prodrugs and their metabolites were determined on a Waters HPLC system (Waters, Inc., Milford, MA, USA). The HPLC system consisted of two Waters pumps (model 515), a Waters autosampler (WISP model 712) and a Waters 996 photodiode array UV detector controlled by Waters Millennium^®^ 32 software (version 3.0.1). Samples were resolved on a Waters Xterra C_18_ reverse-phase column (5 µm, 4.6 x 250 mm) equipped with a guard column. The mobile phase consisted of 0.1% TFA/water (Solvent A) and 0.1% TFA/ACN (Solvent B) with the solvent B gradient changing from 0-56% at a rate of 2%/minute during a 28 minute run. Prodrugs, their metabolites, and their parent drugs were easily separated and identified. Standard curves generated for each prodrug and their parent drugs were utilized for quantitation of integrated area under peaks. The detection wavelength was 254 nm and spectra were acquired in the 220-380 nm range.

### [^3^H]Gly-Sar Uptake Inhibition

Caco-2 cells at nine days post-seeding, and AsPC-1 cells at four days post-seeding were incubated with 10 µmol/L Gly-Sar (9.98 µmol/L Gly-Sar and 0.02 µmol/L [3H]Gly-Sar) along with various prodrug concentrations (5–0.05 mmol/L) for 30 minutes. The cells were washed three times with ice-cold PBS and solubilized with 10 mL of scintillation cocktail (ScintiVerse^®^, Fisher Scientific, St.Louis, MO, USA). The amount of cell-associated radioactivity was determined by scintillation counting (Beckman LS-9000, Beckman Instruments, Fullerton, CA, USA). The half maximal inhibitory concentration (IC_50_) values were determined using nonlinear data fitting (GraphPad Prism version 3.0).

### Caco-2 permeability study

Caco-2 cell monolayers were grown on collagen-coated polytetrafluoroethylene membrane for 21 to 24 days. Transepithelial electrical resistance (TEER) was monitored and values of 240-280 Ω/cm^2^ (total growth area was 4.67 cm^2^) was used in the study. Apical and basolateral sides of transwell inserts were washed with MES (pH 6.0) and HEPES (pH 7.4), respectively. Fresh MES and HEPES buffers were reapplied to transwell inserts and incubated at 37°C for 15 minutes. Each drug was individually tested from freshly prepared solutions in MES buffer (0.1 mM, total 1.5 mL). The solution was placed in the donor chamber, while the receiver chamber was filled with HEPES buffer (total 2.5 mL). Sampling from the receiver chamber was conducted up to a period of 2 hours at time intervals of 15, 30, 45, 60, 75, 90, and 120 minutes, at 37°C and replaced with an equal volume of fresh HEPES buffer to maintain sink conditions in the receiver chamber. All samples were immediately acidified with 0.1% TFA and analyzed by HPLC.

### Cell Proliferation Assays

Cell proliferation studies were conducted with MDCK cells and MDCK cells that express hPEPT1 (MDCK/hPEPT1). The cells were seeded into 96-well plates at 125,000 cells per well and allowed to attach/grow for 24 hours before drug solutions were added. The culture medium (DMEM + 10% fetal bovine serum) was removed and the cells were gently washed once with sterile pH 6.0 uptake buffer. Floxuridine and floxuridine prodrugs were 2-fold serially diluted in pH 6.0 uptake buffer from 4 to 0.25 mmol/L. Buffer alone was used as 100% viability control. The wash buffer was removed and 30 µL drug solution per well was added and incubated at 37°C for 2 hours in the cell incubator. After this time, the drug solutions were removed and the cells were again gently washed twice with sterile uptake buffer. The culture medium was then added to each well after washing. The cells were allowed to recover for 24 hours before evaluating cell viability via 2,3-bis[2-methoxy-4-nitro-5-sulfophenyl]-2H-tetrazolium-5-carboxanilide inner salt (XTT) assays. A mixture (30 µL) containing XTT (1 mg/mL) in sterile DMEM without phenol red and phenazine methosulfate (*N*-methyldibenzopyrazine methyl sulfate in sterile PBS, 0.383 mg/mL) reagents were added to the cells and incubated at 37°C for 1 hour, after which the absorbance at 450 nm was read. The concentrations required to inhibit cell growth by 50% (GI_50_) were calculated using GraphPad Prism version 3.0 by nonlinear data fitting.

## References

[1] Heidelberger C., Chaudhuri N.K., Danneberg P., Mooren D., Griesbach L., Duschinsky R., Schnitzer R.J., Pleven E., Scheiner J. (1957). Fluorinated pyrimidines, a new class of tumour-inhibitory compounds. Nature.

[2] Cohen AM., Minsky B. D., Schilsky R. L.  (1993). Cancer, Principles and Practices in Oncology.

[3] Grem J.L. (2000). 5-Fluorouracil: forty-plus and still ticking. A review of its preclinical and clinical development. Invest. New Drugs.

[4] Willmore E., Durkacz B.W. (1993). Cytotoxic mechanisms of 5-fluoropyrimidines. Relationships with poly(ADP-ribose) polymerase activity, DNA strand breakage and incorporation into nucleic acids. Biochem. Pharmacol..

[5] Parker W.B., Cheng Y.C. (1990). Metabolism and mechanism of action of 5-fluorouracil. Pharmacol. Ther..

[6] van Laar J.A., Rustum Y.M., Ackland S.P., van Groeningen C.J., Peters G.J. (1998). Comparison of 5-fluoro-2'-deoxyuridine with 5-fluorouracil and their role in the treatment of colorectal cancer. Eur. J. Cancer..

[7] Shibamoto Y., Tachi Y., Tanabe K., Hatta H., Nishimoto S. (2004). In vitro and in vivo evaluation of novel antitumor prodrugs of 5-fluoro-2'-deoxyuridine activated by hypoxic irradiation. Int. J. Radiat. Oncol. Biol. Phys..

[8] Yamada M., Nakagawa H., Fukushima M., Shimizu K., Hayakawa T Ikenaka K. (1998). In vitro study on intrathecal use of 5-fluoro-2'-deoxyuridine (FdUrd) for meningeal dissemination of malignant brain tumors. J. Neurooncol..

[9] Laskin J.D., Evans R.M., Slocum H.K., Burke D., Hakala M.T. (1979). Basis for natural variation in sensitivity to 5-fluorouracil in mouse and human cells in culture. Cancer. Res..

[10] Birnie G.D., Kroeger H., Heidelberger C. (1963). Studies Of Fluorinated Pyrimidines. Xviii. The Degradation Of 5-Fluoro-2'-Deoxyuridine And Related Compounds By Nucleoside Phosphorylase. Biochemistry.

[11] Kim I., Chu X.Y., Kim S., Provoda C.J., Lee K.D., Amidon G.L. (2003). Identification of a human valacyclovirase: biphenyl hydrolase-like protein as valacyclovir hydrolase. J. Biol. Chem..

[12] Kim I., Song X., Vig B.S., Mittal S., Shin H.C., Lorenzi P.J., Amidon G.L. (2004). A novel nucleoside prodrug-activating enzyme: substrate specificity of biphenyl hydrolase-like protein. Mol. Pharm..

[13] Landowski C.P., Lorenzi P.L., Song X., Amidon G.L. (2006). Nucleoside ester prodrug substrate specificity of liver carboxylesterase. J. Pharmacol. Exp. Ther..

[14] Han H.K., Oh D.M., Amidon G.L. (1998). Cellular uptake mechanism of amino acid ester prodrugs in Caco-2/hPEPT1 cells overexpressing a human peptide transporter. Pharm. Res..

[15] Landowski C.P., Vig B.S., Song X., Amidon G.L. (2005). Targeted delivery to PEPT1-overexpressing cells, acidic, basic, and secondary floxuridine amino acid ester prodrugs. Mol. Cancer Ther..

[16] Anand B.S., Katragadda S., Mitra A.K. (2004). Pharmacokinetics of novel dipeptide ester prodrugs of acyclovir after oral administration: intestinal absorption and liver metabolism. J. Pharmacol. Exp. Ther..

[17] Anand B.S., Patel J., Mitra A.K. (2003). Interactions of the dipeptide ester prodrugs of acyclovir with the intestinal oligopeptide transporter: competitive inhibition of glycylsarcosine transport in human intestinal cell line-Caco-2. J. Pharmacol. Exp. Ther..

[18] Meredith D., Temple C.S., Guha N., Sword C.J., Boyd C.A., Collier I.D., Morgan K.M., Bailey P.D. (2000). Modified amino acids and peptides as substrates for the intestinal peptide transporter PepT1. Eur. J. Biochem..

[19] Surendran N., Covitz K.M., Han H., Sadee W., Oh D.M., Amidon G.L., Williamson R.M., Bigge C.F., Stewart B.H. (1999). Evidence for overlapping substrate specificity between large neutral amino acid (LNAA) and dipeptide (hPEPT1) transporters for PD 158473, an NMDA antagonist. Pharm. Res..

[20] Wenzel U., Thwaites D.T., Daniel H. (1995). Stereoselective uptake of beta-lactam antibiotics by the intestinal peptide transporter. Br. J. Pharmacol..

[21] Nielsen C.U., Andersen R., Brodin B., Frokjaer S., Taub M.E., Steffansen B. (2001). Dipeptide model prodrugs for the intestinal oligopeptide transporter. Affinity for and transport via hPepT1 in the human intestinal Caco-2 cell line. J. Control. Release..

[22] Satake M., Enjoh M., Nakamura Y., Takano T., Kawamura Y., Arai S., Shimizu M. (2002). Transepithelial transport of the bioactive tripeptide, Val-Pro-Pro, in human intestinal Caco-2 cell monolayers. Biosci. Biotechnol. Biochem..

[23] Wenzel U., Gebert I., Weintraut H., Weber W.M., Clauss W., Daniel H. (1996). Transport characteristics of differently charged cephalosporin antibiotics in oocytes expressing the cloned intestinal peptide transporter PepT1 and in human intestinal Caco-2 cells. J. Pharmacol. Exp. Ther..

[24] Han H., de Vrueh R.L., Rhie J.K., Covitz K.M., Smith P.L., Lee C.P., Oh D.M., Sadee W., Amidon G.L. (1998). 5'-Amino acid esters of antiviral nucleosides, acyclovir, and AZT are absorbed by the intestinal PEPT1 peptide transporter. Pharm. Res..

[25] Weller S., Blum M.R., Doucette M., Burnette T., Cederberg D.M., de Miranda P., Smiley M.L. (1993). Pharmacokinetics of the acyclovir pro-drug valaciclovir after escalating single- and multiple-dose administration to normal volunteers. Clin. Pharmacol. Ther..

[26] Gonzalez D.E., Covitz K.M., Sadee W., Mrsny R.J. (1998). An oligopeptide transporter is expressed at high levels in the pancreatic carcinoma cell lines AsPc-1 and Capan-2. Cancer Res..

[27] Nakanishi T., Tamai I., Takaki A., Tsuji A. (2000). Cancer cell-targeted drug delivery utilizing oligopeptide transport activity. Int. J. Cancer..

[28] Friedrichsen G.M., Chen W., Begtrup M., Lee C.P., Smith P.L., Borchardt R.T. (2002). Synthesis of analogs of L-valacyclovir and determination of their substrate activity for the oligopeptide transporter in Caco-2 cells. Eur. J. Pharm. Sci..

[29] Guo A., Hu P., Balimane P.V., Leibach F.H., Sinko P.J. (1999). Interactions of a nonpeptidic drug, valacyclovir, with the human intestinal peptide transporter (hPEPT1) expressed in a mammalian cell line. J. Pharmacol. Exp. Ther..

[30] Landowski C.P., Sun D., Foster D.R., Menon S.S., Barnett J.L., Welage L.S., Ramachandran C., Amidon G.L. (2003). Gene expression in the human intestine and correlation with oral valacyclovir pharmacokinetic parameters. J. Pharmacol. Exp. Ther..

[31] Phan D.D., Chin-Hong P., Lin E.T., Anderle P., Sadee W., Guglielmo B.J. (2003). Intra- and interindividual variabilities of valacyclovir oral bioavailability and effect of coadministration of an hPEPT1 inhibitor. Antimicrob. Agents Chemother..

[32] Umapathy N.S., Ganapathy V., Ganapathy M.E. (2004). Transport of amino acid esters and the amino-acid-based prodrug valganciclovir by the amino acid transporter ATB(0,+). Pharm. Res..

[33] Hu M., Subramanian P., Mosberg H.I., Amidon G.L. (1989). Use of the peptide carrier system to improve the intestinal absorption of L-alpha-methyldopa: carrier kinetics, intestinal permeabilities, and in vitro hydrolysis of dipeptidyl derivatives of L-alpha-methyldopa. Pharm. Res..

[34] Anand B.S., Dey S., Mitra A.K. (2002). Current prodrug strategies via membrane transporters/receptors. Expert. Opin. Biol. Ther..

[35] Ganapathy M.E., Huang W., Wang H., Ganapathy V., Leibach F.H. (1998). Valacyclovir: a substrate for the intestinal and renal peptide transporters PEPT1 and PEPT2. Biochem. Biophys. Res. Commun..

[36] Eriksson A.H., Elm P.L., Begtrup M., Nielsen R., Steffansen B., Brodin B. (2005). hPEPT1 affinity and translocation of selected Gln-Sar and Glu-Sar dipeptide derivatives. Mol. Pharm..

[37] Li J., Tamura K., Lee C.P., Smith P.L., Borchardt R.T., Hidalgo I.J. (1998). Structure-affinity relationships of Val-Val and Val-Val-Val stereoisomers with the apical oligopeptide transporter in human intestinal Caco-2 cells. J. Drug. Target..

[38] Tamura K., Bhatnagar P.K., Takata J.S., Lee C.P., Smith P.L., Borchardt R.T. (1996). Metabolism, uptake, and transepithelial transport of the diastereomers of Val-Val in the human intestinal cell line, Caco-2. Pharm. Res..

[39] Vabeno J., Lejon T., Nielsen C.U., Steffansen B., Chen W., Ouyang H., Borchardt R.T. (2004). Phe-Gly dipeptidomimetics designed for the di-/tripeptide transporters PEPT1 and PEPT2: synthesis and biological investigations. J. Med. Chem..

[40] Landowski C.P., Song X., Lorenzi P.L., Hilfinger J.M., Amidon G.L. (2005). Floxuridine amino Acid ester prodrugs: enhancing Caco-2 permeability and resistance to glycosidic bond metabolism. Pharm. Res..

[41] Lorenzi P.L., Landowski C.P., Song X., Borysko K.Z., Breitenbach J.M., Kim J.S., Hilfinger J.M., Townsend L.B., Drach J.C., Amidon G.L. (2005). Amino acid ester prodrugs of 2-bromo-5,6-dichloro-1-(beta-D-ribofuranosyl)benzimidazole enhance metabolic stability in vitro and in vivo. J. Pharmacol. Exp. Ther..

[42] Song X., Vig B.S., Lorenzi P.L., Drach J.C., Townsend L.B., Amidon G.L. (2005). Amino acid ester prodrugs of the antiviral agent 2-bromo-5,6-dichloro-1-(beta-D-ribofuranosyl)benzimidazole as potential substrates of hPEPT1 transporter. J. Med. Chem..

[43] Vig B.S., Lorenzi P.J., Mittal S., Landowski C.P., Shin H.C., Mosberg H.I., Hilfinger J.M., Amidon G.L. (2003). Amino acid ester prodrugs of floxuridine: synthesis and effects of structure, stereochemistry, and site of esterification on the rate of hydrolysis. Pharm. Res..

[44] Daniel H., Morse E.L., Adibi S.A. (1992). Determinants of substrate affinity for the oligopeptide/H^+^ symporter in the renal brush border membrane. J. Biol. Chem..

[45] Fang G., Konings W.N., Poolman B. (2000). Kinetics and substrate specificity of membrane-reconstituted peptide transporter DtpT of Lactococcus lactis. J. Bacteriol..

